# Association of Children’s Urinary CC16 Levels with Arsenic Concentrations in Multiple Environmental Media

**DOI:** 10.3390/ijerph13050521

**Published:** 2016-05-23

**Authors:** Paloma I. Beamer, Walter T. Klimecki, Miranda Loh, Yoshira Ornelas Van Horne, Anastasia J. Sugeng, Nathan Lothrop, Dean Billheimer, Stefano Guerra, Robert Clark Lantz, Robert A. Canales, Fernando D. Martinez

**Affiliations:** 1 Asthma and Airways Disease Research Center, University of Arizona, 1501 N. Campbell Ave., Tucson, AZ 85724, USA; dean.billheimer@arizona.edu (D.B.); stefano@email.arizona.edu (S.G.); fernando@arc.arizona.edu (F.D.M.); 2 Mel and Enid Zuckerman College of Public Health, University of Arizona, 1295 N. Martin Ave., Tucson, AZ 85724, USA; klimecki@pharmacy.arizona.edu (W.T.K.); Miranda.loh@iom-world.org (M.L.); yornelas@email.arizona.edu (Y.O.V.H.); asugeng@email.arizona.edu (A.J.S.); lothrop@email.arizona.edu (N.L.); lantz@email.arizona.edu (R.C.L.); rcanales@email.arizona.edu (R.A.C.); 3 Department of Chemical and Environmental Engineering, University of Arizona, 1133 E. James E. Rogers Way, Tucson, AZ 85721, USA; 4 Department of Pharmacology and Toxicology, College of Pharmacy, University of Arizona, P.O. Box 210207, Tucson, AZ 85724, USA; 5 Institute of Occupational Medicine, Research Avenue North, Riccarton, Edinburgh EH14 4AP, UK; 6 Department of Cellular and Molecular Medicine, University of Arizona, P.O. Box 245044, Tucson, AZ 85724, USA

**Keywords:** children, arsenic, respiratory health, CC16, uteroglobulin, multi-route exposure assessment, soil, drinking water

## Abstract

Arsenic exposure has been associated with decreased club cell secretory protein (CC16) levels in adults. Further, both arsenic exposure and decreased levels of CC16 in childhood have been associated with decreased adult lung function. Our objective was to determine if urinary CC16 levels in children are associated with arsenic concentrations in environmental media collected from their homes. Yard soil, house dust, and tap water were taken from 34 homes. Urine and toenail samples were collected from 68 children. All concentrations were natural log-transformed prior to data analysis. There were associations between urinary CC16 and arsenic concentration in soil (*b* = −0.43, *p* = 0.001, *R*^2^ = 0.08), water (*b* = −0.22, *p* = 0.07, *R*^2^ = 0.03), house dust (*b* = −0.37, *p* = 0.07, *R*^2^ = 0.04), and dust loading (*b* = −0.21, *p* = 0.04, *R*^2^ = 0.04). In multiple analyses, only the concentration of arsenic in soil was associated with urinary CC16 levels (*b* = −0.42, *p* = 0.02, *R*^2^ = 0.14 (full model)) after accounting for other factors. The association between urinary CC16 and soil arsenic may suggest that localized arsenic exposure in the lungs could damage the airway epithelium and predispose children for diminished lung function. Future work to assess this possible mechanism should examine potential associations between airborne arsenic exposures, CC16 levels, lung function, and other possible confounders in children in arsenic-impacted communities.

## 1. Introduction

In the United States (US) and Canada, chronic obstructive pulmonary disease (COPD) is the second leading cause of disability-adjusted life years, while asthma is the 22nd leading cause [1]. Childhood asthma and related wheezing disorders remain the most common chronic diseases in US children [2]. Evidence from multiple birth cohorts has established that respiratory and immune alterations present soon after birth and/or by the preschool years are predisposing factors for development of asthma and airflow limitation in adulthood [3]. This indicates that there is a critical window of susceptibility in early life or *in utero* during which environmental exposures may disrupt normal development resulting in early respiratory impairment. If biomarkers can be identified, which are predictive of long-term clinical consequences and can be readily measured in early childhood during this critical window, early-life interventions could be developed to prevent potential long-term health effects in high-risk children.

Club (formerly Clara) cell secretory protein, referred to as CC16, is one such potential biomarker. CC16 is a homodimeric pneumoprotein with anti-inflammatory and anti-toxicant properties [4,5,6]. CC16 is primarily produced by club cells in the distal airways, but can be measured in circulation [4,5,6]. There is a complex relationship between circulating CC16 levels and environmental exposures. Perhaps due to epithelial damage in the lung, circulating CC16 levels increase following acute environmental exposures such as smoking a cigarette or firefighting [7,8]. However, overall circulating levels are lower in those with chronic environmental exposures, such as smokers or firefighters, compared to the general population [9,10]. It has been hypothesized that those with chronic exposures may have fewer or less responsive club cells in their lungs [9,10].

Not only are CC16 levels associated with environmental exposures, but circulating CC16 levels may also be predictive of early respiratory impairments. Lower levels of circulating CC16 in adulthood have been associated with lower lung function, greater airflow limitation, and increased mortality, particularly from lung cancer [11,12]. More recently, lower levels of CC16 at six years of age have been associated with decreased lung function by age 16 years among participants from a longitudinal birth cohort (*i.e.*, the Tucson Children’s Respiratory Study (CRS)), and confirmed in a meta-analysis with other birth cohorts [13]. In a cross-sectional study, asthmatic children had lower levels of CC16 in their urine than non-asthmatic children, and the CC16 levels in urine were significantly related to forced vital capacity [14]. Thus, decreased CC16 may be an important biomarker of early life epithelial and airway damage from potentially preventable environmental exposures, suggesting that interventions could be designed to prevent subsequent disease. 

**Figure 1:**
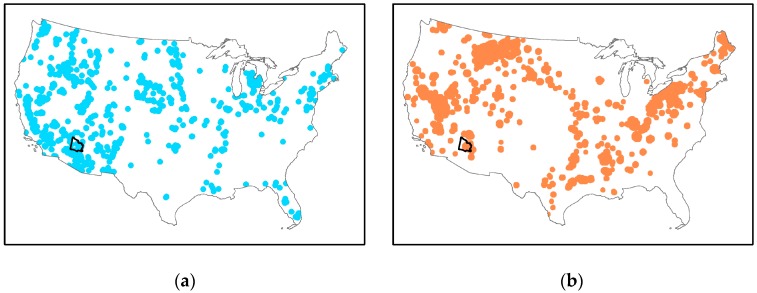
Potential for arsenic exposure in the US. Maps derived from data contained in Focazio *et al.* [16] and Smith *et al.* [17]. Yavapai County, where the study is located, is outlined. (**a**) Well water concentration above US EPA drinking water guideline for arsenic of 10 ppb; (**b**) soil concentration above Arizona Department of Environmental Quality Soil Remediation Level of 10 ppm.

Arsenic ranks first on the US Agency for Toxic Substances and Disease Registry’s 2015 “Priority List of Hazardous Substances” due to its high toxicity and frequency of detection at US Environmental Protection (EPA) National Priority List Sites [15]. Certain regions of the US are disproportionately impacted by arsenic exposure because of their unique geology, leading to higher concentrations of arsenic in groundwater (Figure 1a). Furthermore, many of these same regions have higher concentrations of arsenic in soil (Figure 1b) and therefore, possibly in wind-suspended dust. In these communities, there is great potential for multi-route exposures via multiple environmental media. 

Arsenic is a known carcinogen that is also associated with adverse effects on the respiratory, cardiovascular, immune, endocrine, and neurological systems [18]. Of particular interest is the evidence that suggests that *in utero* or early-life exposures to arsenic via drinking water during critical windows of susceptibility may be related to increased risk of respiratory diseases later in life, such as bronchiectasis [19]. More recently, early-life arsenic exposures have been associated with increased risk of lower respiratory tract illnesses in infants and decreased lung function in children and adults [20,21,22]. These early respiratory impairments are predisposing factors for asthma and COPD in adulthood [3], and may explain associations between arsenic exposure and chronic respiratory disease. Occupational arsenic exposure via air is also associated with decreased CC16 levels [23,24]. In Bangladesh, where arsenic exposures via drinking water are very high, adults without skin lesions but with reduced lung function had higher levels of arsenic exposure and lower levels of circulating CC16 [25]. Thus, CC16 may be a unique biomarker that could be used to assess potential adverse impacts of arsenic on the respiratory system in early life.

Although there is some evidence that high arsenic exposure via multiple routes (*i.e.*, inhalation, ingestion) is associated with decreased CC16 levels in adults [23,24,25], this has not been assessed in children. To date there have been no studies that have examined the association of CC16 levels with concurrent arsenic exposure via multiple routes. We conducted an exploratory study to test for an association between urinary CC16 levels measured in children and arsenic concentrations measured in multiple environmental (*i.e.*, yard soil, house dust, and tap water) and biological (*i.e.*, urine, toenails) matrices.

## 2. Materials and Methods 

### 2.1. Study Population and Recruitment

The Metals Exposure Study in Homes (MESH) took place between 6 June 2012 and 27 June 2013 in the Town of Dewey-Humboldt, Arizona. A detailed description of this study has been provided elsewhere [26]. All houses (*n* = 34) were within seven miles of the Iron King Mine and Humboldt Smelter Site, a Superfund site listed on the US EPA National Priorities List. Households were recruited via mail, door-to-door solicitation, community festivals, and other venues. To be eligible to participate the household had to have at least one child between the ages of 1 and 11 years. However, all children in the household between 1 and 11 years old that could provide a urine sample into a specimen cup were able to participate. Duration of residence in Dewey-Humboldt was not an inclusion criteria for participation. A questionnaire was administered to obtain household information regarding annual income, educational level of the adults, number of smokers, and tap water source, as well as child-specific information regarding residential history, primary drinking water, respiratory disease, and symptoms. Parents completed a four day activity and food log for their children. This was used to estimate the percent of their time spent at home and the percent of their time spent outdoors. By using our dietary logs, we have determined that consumption of apple, cereal, and egg are most strongly associated with urinary levels of inorganic arsenic metabolites in our population compared to our analyses with other foods [27]. As previously explained, recruitment, questionnaire administration, and sample collection were conducted by local community members that were trained by the University of Arizona research team both in the field and in the laboratory [26]. All parents gave their informed consent for their children’s inclusion before they participated in the study. The study was conducted in accordance with the Decalartion of Helsinki, and the protocol was reviewed and approved by the University of Arizona Human Subjects Protection Program (1100000716; approved 7 October 2011).

### 2.2. Sample Collection

This study consisted of two home visits. At the first visit, the consent form was administered and the parents were provided with materials and instructions for collecting the biological samples and the activity and food logs. At the second visit, which occurred two weeks later, the field staff collected the environmental samples and biological samples, administered questionnaires, and verified the completeness of the logs. 

Parents were provided with new sterilized nail clippers for each child. They were instructed to wait two weeks and then clip nails from all ten of their children’s toes and place the clippings in small brown manila envelopes. Parents were also provided with a sterilized urine collection cup. They were instructed to obtain the first morning void and refrigerate the sample until they provided it to the field staff. Parents were instructed to complete the activity and food logs for the four days prior to urine sample collection.

The environmental samples collected at each home included: yard soil, house dust, and tap water. A trowel of yard soil was taken at two locations approximately 3 m perpendicular to each of the four sides of the house. These samples were combined into one plastic bag per house.

House dust samples were taken using a 2.2 horsepower Hoover CH3000 vacuum cleaner (Hoover, Glenwillow, OH, USA) equipped with a pre-weighed X-Cell 100 dust collection sock (Midwestern Filtration, West Chester Township, OH, USA) inserted in the crevice tool of the vacuum, as previously described [26]. At each house, a 1 m^2^ template was laid on the floor in the room where residents spent the majority of their time and vacuumed for two min. This was repeated until approximately 2 g of dust were collected. Hard flooring and carpeting were sampled in proportion to their overall proportion in the room. The collection socks were then placed into a plastic bag. Between uses, the entire vacuum assembly was cleaned inside and out with isopropanol, and the vacuum cleaner bag was verified to be empty. The vacuum was stored and transported in a plastic tub to prevent contamination. The crevice tool was cleaned with isopropanol a second time in the home, prior to insertion of the dust collection sock.

Following a 2-min flush, water samples were collected from the kitchen tap into 15 mL trace-metal free centrifuge tubes. These samples were transported on ice and refrigerated at 4 °C in the field office. 

All samples were transported to the University of Arizona for analysis within two weeks of collection. Urine and water samples were transported on ice. 

### 2.3. Sample Processing

Toenail samples were first sonicated in acetone for 20 min, then five times with ultra-pure water (Type I Clinical Water), and then sonicated again in 1% Triton-X, non-ionic surfactant, for an additional 20 min. Following the sonications, they were rinsed again with ultrapure water and dried in an oven at 60 °C for 12 h before being weighed on an analytical balance. Toenails were soaked overnight (*i.e.*, 12 h) in 2 mL of reagent grade concentrated nitric acid before being digested in a microwave (Mars Xpress, CEM Corporation, Matthews, NC, USA). The microwave was set at 400 W with 75% power, with a 10 min ramp up to 105 °C, and held at that temperature for 15 min. Samples were cooled before being transferred to centrifuge tubes and diluted to 10 mL with nitric acid. A certified reference material (INSPQ/Toxicologie QMEQAS10H-02, cheveux/hair) was used for quality control.

Urine samples were stored at −20 °C until analysis. Once the samples were thawed, the specific gravity of urine samples was determined using a refractometer (TS Meter Handheld Goldberg Series, Reichert Analytical Instruments, Depew, NY, USA). Samples were filtered at 0.45 µm using nylon microcentrifuge vials in bench top refrigerated centrifuge (Beckman Model 20R with 36-place BioSafe rotor, Beckman Coulter, Brea, CA, USA) prechilled to 4 °C and centrifuged for 4 min at 14,000 rpm. A portion of the filtrate (0.3 mL) was transferred to a dilution tube and combined with 2.7 mL of speciation buffer (ammonium carbonate eluent plus EDTA).

Soil and dust samples were dried in an oven at 105–110 °C for 24 h. The entire dust or soil sample was homogenized and sieved using an electrical shaker (Ro-tap, Tyler, Mentor, OH, USA). 500 mg of each soil and dust sample, with particle size <63 µm, was microwave digested in 10 mL of concentrated reagent grade nitric acid according to EPA Method 3051.

Tap water samples were preserved within two weeks of collection with enough concentrated reagent grade nitric acid to lower the pH to less than 2.0 units. Before water samples were sent for analysis, they were confirmed to have a turbidity <1 Nephelometric Turbidity Unit (NTU) with an Orion AQ4500 Turbidity Metter (Thermo Scientific, Waltham, MA, USA).

### 2.4. Laboratory Analyses

The Arizona Laboratory for Emerging Contaminants (ALEC) analyzed the concentration of arsenic in the urine, water, digested toenails, soil, and dust using inductively coupled plasma mass spectrometry (ICP-MS) (7700x ICP-MS, Agilent Technologies, Santa Clara, CA, USA). The analytical minimum detection limit was 0.5 µg/L for arsenic. Ultra-pure water (Type I Clinical Water) and nitric acid blanks were submitted along with the samples.

Calibration standards were prepared from multi-element stock solutions purchased from AccuStandard (New Haven, CT, USA). Stock solutions were diluted in 1% nitric acid to provide a working calibration curve of at least five points. Samples were also diluted with 1% nitric acid until their response was determined to be within the calibration range. For analytical quality control, a check solution (from an independent source and comparable to a low-to-midrange standard) was analyzed after the calibration and before each sample set. Also, a NIST sample (NIST 1643e Trace metals in water) was included at the beginning and end of each sample set to assess quality control on the dissolved metals in solution. A mid-range standard was analyzed after every 10 samples and at the end of the run, and the results were within 25% of the expected value. Elemental internal standards (Rh, In, and Ga) were added to both standards and samples prior to analysis. All analytical measurements were performed in triplicate with an average relative standard deviation of 3%.

Arsenic speciation in urine was conducted using high performance liquid chromatography with a method adapted from Milstein *et al.* [28]. The HPLC system consisted of an Agilent 1100 HPLC with Hamilton PRP-X100 column (10 µm, 150 × 4.1 mm) and guard cartridge. The mobile phase was ammonium carbonate (pH = 8.75) with 2% methanol in a gradient (10 mM for 2 min, ramp to 50 mM over 1 min, and held for 9 min). The column temperature was maintained at 30 °C and samples were kept at 4 °C in a temperature-controlled auto-sampler. The ICP-MS with a Micromist nebulizer (Glass Expansion) served as the detector. The species measured included: arsenite (As(+3)), arsenate (As(+5)), monomethylarsonate (MMA), dimethylarsinate (DMA), arsenobetaine (AsB), and arsenocholine (AsC)). 

The concentration of CC16 in urine was analyzed using a human uteroglobulin quantikine ELISA kit according to manufacture protocol (R and D Systems, Minneapolis, MN, USA). The limit of detection (LOD) was 0.8 ng/mL.

### 2.5. Data Analyses

Prior to analyses, concentration of arsenic in the nitric acid and ultra-pure water blanks was subtracted from the concentration of arsenic in the toenail, soil, dust, and water samples, respectively. Samples with concentrations below the detection limit or less than 3 times the standard deviation of the blanks were assigned a value equal to the minimum detection limit divided by the square root of two [29], prior to subsequent calculations. Only one toenail sample concentration was replaced because it was less than 3 times the standard deviation of the blanks. All other censored values were replaced because they were below the detection limit. The initial sample mass prior to acid digestion was used to calculate the concentration of arsenic in toenail, dust, and soil samples. Samples above the “upper limit of detection” for CC16 were substituted with the highest standard concentration (53 ng/mL) in the linear range. The concentrations of arsenic species and CC16 in urine were corrected for specific gravity [30]. This adjustment was conducted for both censored and measured values. Arsenic dust loading was calculated as the concentration of arsenic in dust multiplied by the mass of dust (<63 µm) per square meter. As environmental concentrations are log-normally distributed [31], all data were log-transformed prior to analysis. 

We used simple linear regression to assess the association between urinary CC16 concentrations and our continuous and categorical variables. Since there are multiple children in many households, we used the robust cluster estimator of variance to account for potential clustering by household. We have previously demonstrated that there are correlations between the concentration of arsenic in the multiple environmental media and in the biomarkers [26]. Therefore, we calculated partial correlations to understand how these multiple variables related to each other and to our outcome of interest (*i.e.*, urinary CC16). Variables that were partially correlated with urinary CC16 and variables that were partially correlated with those variables, themselves, were included in the final multiple regression model. To assess multi-collinearity, we calculated the variance inflation factor (VIF) for the final adjusted models. A VIF >5 for any variable may indicate cause for concern [32]. We repeated all of our analyses, with the subset of samples with a specific gravity ≥1.01. Data analysis was performed using STATA 13 (StataCorp, College Station, TX, USA).

## 3. Results

We recruited 70 children across 34 households with 22 households having more than one child participate [26]. One four-year-old male and a one-year-old female did not provide urine samples and have not been included in this analysis. CC16 was detected in 41% of the urine samples, however, the detectable concentrations had a very large range spanning several orders of magnitude (Table 1). Following specific gravity adjustment, the urinary CC16 values were log-normally distributed.

Of the children that provided urine samples, 50% were male and the median age was six years (range: 1–11 years) (Table 2 and Table 3). There was no difference in the concentration of urinary CC16 by gender and there was no association between CC16 concentration and age. Similarly, there were no differences in urinary concentration of CC16 by the highest level of education attainment of household adults, the number of smokers, the number that smoke inside the home, or respiratory symptoms and disease of the children (Table 2). There was also no significant difference in urinary CC16 by the source of household tap water, type of water primarily drunken by the child, or the frequency that the child placed non-food items in their mouth. Annual household income was not associated with urinary CC16 levels (Table 3). There were no associations with the time the child had lived at their current residence or the distance from their house to the mine tailings. Similarly, there was no association with percentage of time the child spent at home or outdoors, or their dietary history over the previous four days (Table 3).

Arsenic was detected in 100% of the urine samples (Table 4), and the most common species detected were As(+5), DMA, and MMA (Table 2). AsC was only detected in two samples and not included in further analyses. Arsenic was only detected in 60% of the toenails. Only one toenail sample concentration was less than 3 times the SD of the blanks, and was treated as a censored value. Although all censored values were assigned the same value for the concentration of arsenic in the acid digestion, the calculation of toenail concentration included normalization by the mass of toenails digested. This resulted in a log-normal distribution for toenail concentration. However, arsenic was detected in 100% of the yard soil, house dust, and tap water samples (Table 5).

**Figure 2:**
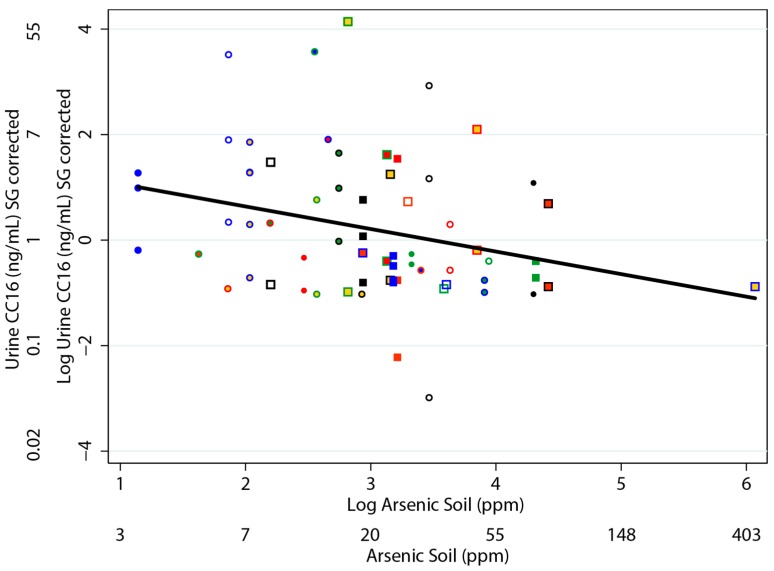
Relationship between concentration of CC16 in urine and arsenic in soil. Individual households are indicated by the same shape and color scheme. Both variables have been natural-log transformed.

Among the simple regressions that we performed, the concentration of CC16 in urine was most strongly (and negatively) associated with the concentration of arsenic in the soil and the arsenic dust loading (Table 6). Similarly, there was a negative relationship between the concentration of CC16 in the urine and with the concentration of arsenic in toenails, house dust, and tap water. However, these relationships were not statistically significant at α = 0.05. Although not significant, of all the arsenic species, the concentration of CC16 in urine was most strongly negatively associated with the concentration of As(+3) in urine compared to our analyses with the other urinary arsenic species. There was no evidence of an association between the concentration of CC16 in urine and the concentrations of the other arsenic species. Analyses restricted to children with a urinary specific gravity of ≥1.01 provided similar results. The relationship between the concentration of urinary CC16 and the concentration of arsenic in soil is provided in Figure 2. We also conducted a sensitivity analysis without the highest arsenic soil concentration, and obtained consistent results (*b* = −0.44, *p* = 0.005).

**Figure 3:**
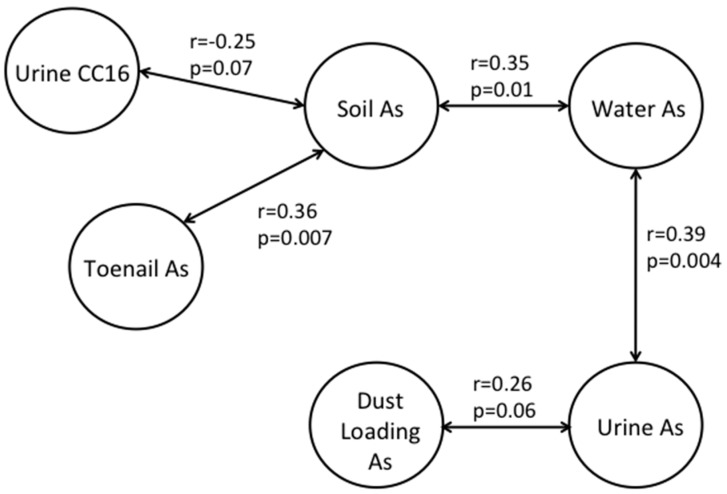
Partial correlations between urinary CC16 and the concentration of arsenic in soil, dust, toenails, urine, and water. Arrows are used to depict partial correlations with a *p* ≤ 0.10.

Since we have previously demonstrated associations between our biomarkers of exposure (*i.e.*, urinary inorganic-related arsenic species and toenail arsenic) and the concentration of arsenic in multiple environmental media [26], we examined the partial correlations between these factors and with the concentration of CC16 in urine. Arsenic dust loading, which was calculated directly from the concentration of arsenic in dust, had a statistically significant association with urinary CC16, whereas the concentration of arsenic in dust did not. Therefore, it was the only dust variable considered in the partial correlations. The partial correlations with a *p* ≤ 0.10 are depicted in Figure 3. Urinary CC16 was partially negatively correlated with the concentration of arsenic in soil. The concentration of arsenic in soil was positively correlated with the concentration of arsenic in water and toenails. This indicates that the associations between urinary CC16 and arsenic in water and toenails are mostly likely because of the correlations between soil and those media. Similarly, the concentration of arsenic in toenails likely represents long-term exposures to arsenic in the indoor environment, represented by the concentration of arsenic in soil. The concentration of urinary inorganic-related arsenic species was associated with the concentration of arsenic in water and the arsenic dust loading. There are likely to be other indoor sources such as smoking or cooking, as well as finer outdoor particles that contribute to the indoor house dust levels. Therefore, arsenic may be present in different forms or mineral structures in house dust and in yard soil. This may explain the stronger association between urinary CC16 and arsenic in soil compared to arsenic in dust in our current study, even though children spend more of their times indoors (Table 3). Similarly, this may also explain the stronger association between urinary CC16 and As(+3) compared to the other arsenic species in urine that we evaluated in the current study. The association between arsenic concentration in toenails and soil may indicate the effect of external dust arsenic contamination on toenails despite the washing procedures before analysis. Arsenic binds strongly to sulfhydryl groups in keratin and may be difficult to remove without also removing some internal arsenic deposition. 

We included the variables that exhibited partial correlations with urinary CC16 and/or each other in a multiple regression. After controlling for the concentration of arsenic in dust loading, toenails, water and inorganic-related urinary arsenic, the concentration of arsenic in soil was negatively correlated with the concentration of CC16 in urine (Table 7). These results were consistent when analyses were restricted to those children with a specific gravity of ≥1.01.

## 4. Discussion

To the best of our knowledge, this study is the first to document a negative association between urinary CC16 levels and arsenic exposure in children. In our study, urinary CC16 levels in children were most strongly negatively associated with the concentration of arsenic in their yard soil and exhibited weaker associations with the concentration of arsenic in tap water and house dust. It is also the first study to demonstrate that diminished CC16 levels may be possibly associated with exposure to arsenic via multiple routes. Our findings indicate that CC16 may be a sensitive biomarker to assess potential early-life damage to the respiratory system associated with arsenic exposure via environmental media such as soil.

Our results confirm those from the relatively few studies that have assessed the relationship between arsenic exposure and CC16 levels. These previous studies each focused primarily on one exposure route and environmental medium. Among copper smelter workers, arsenic concentrations in personal air samples and not total urinary arsenic concentrations, were negatively associated with CC16 levels in serum [23]. In a subsequent study of copper smelter workers, diminished serum levels of CC16 were associated with urinary concentrations of As(+3) and As(+5) but not total arsenic or the methylated metabolites [24]. Collectively, these results indicate that localized inorganic arsenic exposure in the lungs may have a greater impact on CC16 levels than systemic arsenic exposures. In the current study, we also found that CC16 levels were more likely to be associated with external environmental measurements of arsenic (*i.e.*, soil), than urinary arsenic levels. 

In Bangladesh, where very high levels of arsenic in drinking water are endemic, decreased levels of CC16 in adults were more strongly associated with urinary arsenic concentrations than with the concentration of arsenic in drinking water [25]. The authors of that study speculated that this may be because they only sampled water from one well for each individual and that urinary arsenic exposure may be a better indicator of aggregate arsenic exposures via multiple sources. Cumulative drinking water exposure, calculated as an individualized metric incorporating multiple wells and exposure durations, also had a stronger association than concurrent drinking water exposure. This may indicate that the timing of arsenic exposure may be important on adult CC16 levels. In contrast, our results demonstrated a stronger association between CC16 levels and concentration of arsenic in soil and in tap water, compared to the associations with urinary arsenic concentrations. However, we collected our tap water samples at the household level, which likely provided us with a better measure of an individual’s arsenic exposure via drinking water.

Our results are novel because unlike previous studies conducted among highly exposed adults [23,24,25], we document these associations among children and at much lower arsenic concentrations, which are common among rural US communities. While arsenic exposures in our study are lower than those observed in other CC16 studies, they are higher than those of the general US population [26], and are comparable to values in other studies showing associations between arsenic exposures and adverse respiratory health effects. For example, in our Arizona study, 88% of the children (60/68) had urinary total arsenic levels ≥6 µg/L; a level which has been associated with nearly two times higher odds of pneumonia [33]. Lower respiratory tract infections were associated with maternal drinking water exposure in a prospective birth cohort in New Hampshire with similar water exposures [21]. Furthermore, in a study of adults from a different legacy-mining community in Arizona, comparable levels of arsenic exposure were positively associated with biomarkers measured in sputum, which are sensitive indicators of increased airway inflammation [34]. In the US, approximately 13 million people drink water from unregulated private wells that contain arsenic concentrations above the US EPA Maximum Contaminant Level (MCL) of 10 ppb [35], and approximately another 11 million people drink water from regulated water utilities that have reported arsenic concentrations above the MCL [36]. Thus, approximately 8% of the total US population (318 million people) is exposed to arsenic concentrations in their drinking water that could cause adverse respiratory health effects.

Although much of the focus is on health effects of arsenic from drinking water, in addition to our study, other studies have demonstrated associations between arsenic concentrations in soil or air and health outcomes in children, including leukemia, neurological effects, and biomarkers of genotoxicity [37,38,39,40,41,42]. Although a few of these studies were located in communities where soil concentrations were much higher than our study [37,38], several of these associations were determined in communities where arsenic concentrations in soil and air were comparable or even much lower than in our community [39,41,42]. Although there are several limitations to these studies, given this growing list of health effects potentially associated with arsenic exposure via soil or air and the relative importance of this route in certain communities [43], future studies should be conducted to further assess these associations. These studies should be conducted with much larger populations so that potential confounders can be more thoroughly explored. This is particularly relevant given the importance of early-life arsenic exposure on life-long health, and that arsenic contaminated soil can be resuspended, inhaled, and deposited directly in the respiratory tract.

Occasional high wind events are known to resuspend the Iron King Mine tailings and cause high particulate matter concentrations in the Dewey-Humboldt region [44]. It is possible that particulate matter alone, regardless of arsenic concentration, in such an arid and dusty environment could be a factor related to respiratory injury. Following acute events, one might see an increase in urinary CC16 and with prolonged long-term exposure this may have been the cause of lower CC16 levels in these children. However, we did not observe an association between CC16 levels and proximity to tailings (Table 3), the primary source of wind-blown dust. Future studies should be conducted that examine associations with airborne arsenic levels as particulate matter and other potential airborne contaminants.

Given the exploratory nature of this study, there are limitations that may impact interpretation or generalizability of the findings. One limitation is that we measured CC16 in urine and not in serum, as is more common. Although there is some concern regarding measurement of CC16 in urine because of potential contribution from the urogenital system, it has been demonstrated that this is not likely an issue for females or prepubescent males because the majority of urogenital CC16 arises from the prostate [45]. More studies are needed that compare CC16 levels in serum with CC16 levels in urine, particularly for children, as these methods are less obtrusive and more acceptable to this vulnerable population.

One limitation of the study is a high number of samples below the LOD for the CC16 assay (59%). It is possible that this may be related to the high number of children (*n* = 10) with relatively low urinary specific gravity (<1.01). We, thus, conducted sensitivity analyses, by repeating our analyses with only those children whose urinary specific gravity was ≥1.01 (*n* = 58). Results were consistent between these subset analyses and the full population, although we had less power (Table 6 and Table 7). We assigned samples with concentrations below the limit of detection of less than 3 times the standard deviation of the blanks a value equal to the limit of detection divided by the square root of two. Even though, after adjustment for specific gravity, we had a log-normal distribution of concentrations, assigning all of these censored data the same value, likely decreased the power of our analysis. The urinary levels of CC16 in our study are within the range of what has been reported in the few other studies that have measured CC16 in healthy children’s, as well as in asthmatic children’s, urine [14,46,47]. In the future, studies should use an ELISA kit with a lower limit of detection, as was recently reported by Ma and colleagues [14].

Another limitation is that urine and toenail samples were collected by the parent, or by the child with help of the parent. While this was important for increasing trust, participant compliance, and reducing loss of study subjects, samples were not collected by trained staff. It is possible that this may have increased the potential for contamination. However, increased urinary arsenic was not associated with decreased urinary CC16 levels and it is not likely that lack of trained staff resulted in lower CC16 levels. Toenails were sonicated twice in acetone followed by five rinses with clinical-grade lab water prior to digestion, which likely reduced potential contamination from parental collection of the samples. 

A key limitation is the small sample size of our study, due to the original limited geographic scope (*i.e.*, <5 miles from the Superfund site). This limited our study power, and likely limited our ability to detect associations with urinary CC16. We also conducted 11 different simple regressions (Table 6). We used a Bonferroni correction (α = 0.05/12 = 0.004) to account for multiple comparisons. However, the association between soil arsenic concentration and urinary CC16 levels would still be significant (*p* = 0.001) indicating that there is less chance that this finding is as result of multiple comparisons. We have previously reported that there are high levels of arsenic in soil and well water in our community and that they are likely to be naturally occurring and do not show an association with the legacy mining site [26]. Given the high levels of arsenic in soil and water throughout Yavapai County and in other areas of Arizona (Figure 1), in the future we will expand the geographic scope to determine if our findings are consistent in a larger population of children where potential confounders (Table 2 and Table 3) can be more thoroughly explored.

## 5. Conclusions 

In conclusion, in this exploratory study we determined that lower levels of CC16 in children’s urine may be associated with exposure to arsenic via multiple routes. In particular, we demonstrated that the concentration of arsenic in soil in the area that surrounds a child’s dwelling may have an influence on CC16 is as important as, or even more important than, that of arsenic contained in drinking water. Our findings highlight that CC16 may be a novel biomarker for assessing early damage from arsenic in a child’s environment, even at low-to-moderate levels of exposure. Although many of the associations between arsenic exposure and adverse health effects have been documented in countries where exposures are much higher than in the US, our findings contribute to the growing evidence of potential adverse health effects associated with arsenic exposures at levels commonly encountered among rural US communities, which have been traditionally understudied. Future work should examine potential associations between airborne arsenic exposures, soil arsenic exposures, CC16 levels, and lung function in children, while accounting for potential confounders such as diet, to better understand and confirm these associations.

## Figures and Tables

**Table 1:** Summary of specific gravity and CC16 (ng/mL) in urine collected from children (*n* = 68). Both the raw and specific-gravity adjusted CC16 concentrations are presented.

Analyte	Mean	SD ^1^	Median	IQR ^2^	Range
Specific Gravity	1.020	0.008	1.023	0.012	1.003–1.030
CC16 (raw)	4.1	10.9	ND ^3^	1.3	ND–53.0
CC16 (adjusted)	3.9	9.6	0.8	2.8	0.1–62.9

^1^ SD: standard deviation, ^2^ IQR: interquartile range, ^3^ ND: not detected.

**Table 2:** Summary of participant’s characteristics (*n* = 68) and differences in adjusted urinary CC16 levels by these categories.

Characteristic		*n*	%	*b*	*p*
Gender	Male	34	50	Ref.	
	Female	34	50	−0.39	0.26
Highest Level of Education for Adults in Home	High School	6	9	Ref.	
	Some College	12	17	0.16	0.60
	College	17	25	0.23	0.52
	Graduate Degree	34	49	0.44	0.22
Number of Smokers in Home	0	46	68	Ref.	
	1	16	23	0.25	0.50
	2	6	9	−0.39	0.14
Number of Smokers who Smoke in the Home	0	61	87	Ref.	
	1	3	4	−0.19	0.81
	2	6	9	−0.46	0.43
Source of Tap Water	Municipal (Public) Supply	30	44	Ref.	
	Private Well	38	56	−0.33	0.29
Child’s Primary Drinking Water	Tap	32	51	Ref.	
	Both	15	24	0.27	0.55
	Bottled/Vended	16	25	0.22	0.62
Frequency of Placing Non-Food Items in Mouth	Never	48	71	Ref.	
	Less than once per day	7	10	−0.51	0.21
	About once per day	5	7	−0.96	0.11
	Multiple times per day	8	12	−0.35	0.34
Symptoms/Disease					
Runny/Itchy Nose	Yes	26	38	Ref.	
	No	42	62	−0.19	0.53
Rhinitis/Allergies	Yes	12	18	Ref.	
	No	56	82	−0.15	0.68
MD Diagnosed Asthma	Yes	7	10	Ref.	
	No	61	90	0.04	0.93
Eczema/Atopic Dermatitis	Yes	14	21	Ref.	
	No	54	79	0.12	0.78

Ref.: reference group.

**Table 3:** Summary of continuous characteristics of children who provided a urine sample (*n* = 68), and regression with adjusted urinary CC16.

Characteristic	*n*	Mean	SD ^1^	Median	IQR ^2^	Range	*b*	*p*
Age (years)	68	6.3	2.9	6	5	1–11	0.03	0.56
Annual Household Income ($10,000)	30	77	39	78	41	19–177	0.02	0.54
Time Child has Lived at Current Residence (years)	65	3.7	2.8	4	5	<1–10	−0.08	0.22
Distance to Tailings (km)	34	2.5	2.1	2.1	2.2	0.4–11.2	0.06	0.52
Awake time at home (%)	64	24	20	20	20	0–100	−0.07	0.93
Awake time outside (%)	65	9	7	7	11	0–30	−0.03	0.99
Diet (mg/kg/day)								
Apple	63	1.1	2.3	0	0	0–7.5	0.002	0.92
Cereal	63	0.3	0.27	0.26	0.7	0–1.2	0.04	0.77
Egg	63	0.7	1.1	0	1.5	0–3.6	0.03	0.50

^1^ SD: standard deviation; ^2^ IQR: interquartile range.

**Table 4:** Summary of CC16 and arsenic in urine, and of arsenic in toenails collected from children (*n* = 68). All urine samples were corrected for specific gravity.

Matrix	Units	LOD ^1^	DF ^2^	Mean	SD ^3^	Median	IQR ^4^	Range
Urine								
Total As	µg/L	0.5	100%	18.7	16.6	13.2	13.7	3.1–80.7
As(+3)	µg/L	0.2	57%	1.0	1.0	0.8	1.2	ND–5.4
As(+5)	µg/L	0.1	88%	1.5	4.6	0.6	0.8	ND–37.5
DMA	µg/L	0.1	100%	9.3	8.6	7.5	5.2	1.7–52.5
MMA	µg/L	0.2	88%	1.8	2.1	1.3	1.5	0.1–13.7
Ing. Rel. As ^5^	µg/L		100%	13.4	12.0	11.0	6.2	2.4–72.4
AsB	µg/L	0.1	51%	2.8	8.0	0.3	1.2	ND–44.1
AsC	µg/L	0.1	3%	-	-	ND ^6^		ND–1.4
Toenail								
As	µg/g		60%	1.0	1.4	0.6	0.9	0.1–9.4

^1^ LOD: limit of detection; ^2^ DF: detection frequency; ^3^ SD:standard deviation; ^4^ IQR: interquartile range; ^5^ Ing. Rel. As: inorganic-related arsenic species (sum of As(+3), As(+5), DMA and MMA); ^6^ ND: not detected.

**Table 5:** Summary of arsenic concentration in multiple environmental matrices collected from homes (*n* = 34).

Matrix	Units	DF ^1^	Mean	SD ^2^	Median	IQR ^3^	Range
Soil	µg/g	100%	31.4	53.4	20.9	21.6	3.1–432.5
Dust	µg/g	100%	13.2	9.6	11.4	10.7	1.5–44.1
Dust Load	µg/m^2^	100%	2.01	2.31	1.32	2.39	0.1–11.2
Water	µg/L	100%	17.7	41.3	10.3	12.9	1.6–240.1

^1^ DF: detection frequency; ^2^ SD:standard deviation.

**Table 6:** Simple regressions between the concentration of CC16 in urine (*n* = 68) and the arsenic concentration in biological and environmental samples, clustered by household (*n* = 34). Subset analyses (*n* = 58) are restricted to those with specific gravity ≥1.01 and clustered by household.

Matrix	Analyte	All (*n* = 68)			Specific Gravity ≥1.01 (*n* = 58)		
		*b*	*p*	*R*^2^	*b*	*p*	*R*^2^
Urine	Total As	0.16	0.46	0.01	0.19	0.46	0.01
	As(+3)	−0.19	0.11	0.03	−0.22	0.08	0.04
	As(+5)	0.09	0.23	0.01	−0.07	0.47	0.004
	DMA	−0.14	0.46	0.004	−0.07	0.75	0.001
	MMA	−0.11	0.44	0.01	−0.13	0.40	0.01
	Ing. Rel. As ^1^	−0.08	0.68	0.001	−0.21	0.34	0.01
	AsB	0.01	0.95	0.0001	0.01	0.87	0.001
Toenail		−0.19	0.16	0.03	−0.21	0.12	0.04
Soil		−0.43	0.001	0.08	−0.42	0.007	0.07
Dust		−0.37	0.07	0.04	−0.29	0.22	0.02
Dust Load		−0.21	0.04	0.04	−0.18	0.13	0.03
Water		−0.22	0.07	0.03	−0.19	0.14	0.02

^1^ Ing. Rel. As: inorganic-related arsenic species (sum of As(+3), As(+5), DMA and MMA).

**Table 7:** Multiple regressions between the concentration of CC16 in urine (*n* = 68) with the arsenic concentration in biological and environmental samples, clustered by household (*n* = 34). Subset analyses (*n* = 58) are restricted to those with specific gravity ≥1.01 and clustered by household.

Matrix	VIF	All (*n* = 58) ^1^		Specific Gravity ≥ 1.01 (*n* = 50) ^2^	
		*b*	*p*	*b*	*p*
Toenail	1.45	−0.05	0.74	−0.06	0.71
Soil	1.93	−0.42	0.02	−0.38	0.06
Dust Loading	1.36	−0.09	0.45	−0.06	0.67
Water	1.83	−0.04	0.79	−0.01	0.96
Ing. Rel. As Urine ^3^	1.64	0.23	0.37	0.09	0.81

VIF: variance inflation factor. ^1^ *R*^2^ = 0.14 (full model); ^2^ *R*^2^ = 0.13 (full model); ^3^ Ing. Rel. As: inorganic-related arsenic species in urine (sum of As(+3), As(+5), DMA and MMA).

## Abbreviations

The following abbreviations are used in this manuscript:

ALEC	Arizona Laboratory for Emerging Contaminants
As	arsenic
CC16	club cell secretory protein
COPD	chronic obstructive pulmonary disorder
DF	detection frequency
DMA	dimethylarsinate
ELISA	enzyme-linked immunosorbent assay
HPLC	high performance liquid chromatography
ICP-MS	inductively coupled plasma mass spectrometry
LOD	limit of detection
MCL	maximum contaminant level
MESH	Metals Exposure Study in Homes
MMA	methylarsonate
NTU	Nephelometric Turbidity Unit
OR	odds ratio
US	United States
US EPA	United States Environmental Protection Agency
